# 高三尖杉酯碱联合维奈克拉、阿扎胞苷治疗初诊急性髓系白血病的疗效及安全性初步分析

**DOI:** 10.3760/cma.j.cn121090-20241111-00443

**Published:** 2025-10

**Authors:** 昊 艾, 倩 王, 鸿飞 吴, 青松 尹

**Affiliations:** 郑州大学附属肿瘤医院、河南省肿瘤医院血液科，郑州 450008 Department of Hematology, the Affiliated Cancer Hospital of Zhengzhou University, Zhengzhou 450008, China

**Keywords:** 高三尖杉酯碱, 维奈克拉, 阿扎胞苷, 初诊, 白血病，髓系，急性, Homoharringtonine, Venetoclax, Azacytidine, Leukemia, myeloid, acute

## Abstract

**目的:**

比较高三尖杉酯碱联合维奈克拉、阿扎胞苷（VHA）和VA方案诱导治疗初诊不适合强化疗及老年急性髓系白血病（AML）的疗效及安全性。

**方法:**

回顾性分析2018年9月至2021年7月郑州大学附属肿瘤医院收治的共计59例应用VHA或VA方案治疗的初诊AML患者临床资料，男25例，女34例，中位年龄63岁，其中26例采用VHA方案治疗，33例采用VA方案治疗。比较两组患者总有效率（ORR）、复合缓解率［完全缓解（CR）+ 血细胞计数未完全恢复的完全缓解（CRi）］、微小残留病（MRD）、总生存（OS）率、无复发生存（RFS）率及不良反应。采用Kaplan-Meier法和Cox回归模型进行生存预后的单因素及多因素分析。

**结果:**

VHA组ORR为88.4％（23/26），其中CR 21例、部分缓解（PR）2例；VA组ORR为90.9％（30/33），其中CR 25例、PR 5例，两组疗效差异无统计学意义（*P*＝0.458）。VHA组、VA组1个疗程MRD转阴率分别为73.1％（19/26）和60.6％（20/33），差异无统计学意义（*P*＝0.315）。高危患者中，VHA组、VA组1个疗程复合缓解率分别为78.6％（11/14）和50.0％（5/10），差异无统计学意义（*P*＝0.143）；1个疗程MRD转阴率分别为64.3％（9/14）和20.0％（2/10），差异有统计学意义（*P*＝0.032）。患者的不良反应主要是骨髓抑制、胃肠道反应、粒细胞缺乏期感染，VHA组治疗后Ⅲ～Ⅳ级中性粒细胞减少、血红蛋白减少发生率与VA组差异无统计学意义，Ⅲ～Ⅳ级血小板减少的发生率高于VA组（76.9％对45.5％, *P*＝0.015）。中位随访时间13（1～59）个月，VHA组与VA组1年RFS率分别为69.9％（95％ *CI*：53.1％～92.2％）和55.6％（95％ *CI*：40.1％～77.1％），差异无统计学意义（*P*＝0.305）。1年OS率分别为91.7％（95％ *CI*：77.3％～100％）和58.2％（95％ *CI*：41.7％～81.4％），差异有统计学意义（*P*＝0.024）。高危患者中，VHA组的1年RFS率及OS率均高于VA组（RFS：66.2％对37.5％, *P*＝0.046；OS：85.7％对48.0％, *P*＝0.011），接受异基因造血干细胞移植（allo-HSCT）能够明显提高OS率及RFS率（*P*值分别为0.027、0.047）。多因素分析显示ELN分层、1个疗程MRD转阴为影响RFS的独立预后因素。不同用药方案、1个疗程MRD转阴及是否移植为影响OS的独立预后因素。

**结论:**

VHA方案治疗初诊不适合强化疗及老年AML患者存在临床获益，尤其对于高危患者，序贯allo-HSCT能够获益，且相关不良反应可控。

## Abstract (en)

**Objective:**

To compare the efficacy and safety of two induction regimens, homoharringtonine plus venetoclax and azacitidine（VHA）versus venetoclax and azacitidine（VA）, for newly diagnosed acute myeloid leukemia（AML）patients who are elderly or ineligible for intensive chemotherapy.

**Methods:**

We retrospectively analyzed the clinical data of 59 newly diagnosed AML patients treated with the VHA or VA regimen at Zhengzhou University Affiliated Cancer Hospital from September 2018 to July 2021. The cohort included 25 males and 34 females, with a median age of 63 years. The overall response rate（ORR）, composite complete remission（CRc）rate［CR+CR with incomplete hematologic recovery（CRi）］, minimal residual disease（MRD）negativity rate, overall survival（OS）, relapse-free survival（RFS）, and adverse events were compared between the two groups. Survival was estimated by the Kaplan-Meier method, and prognostic factors were evaluated using univariable and multivariable Cox regression.

**Results:**

At the end of the treatment, the ORR was 88.4％（23/26）in the VHA group［21 CR, 2 partial remissions（PR）］and 90.9％（30/33）in the VA group（25 CR, 5 PR）, with no significant difference between the groups（*P*＝0.458）. The MRD negativity rates after one cycle of induction were 73.1％（19/26）in the VHA group and 60.6％（20/33）in the VA group, respectively（*P*＝0.315）. In the high-risk subgroup, the composite remission rates after one cycle were 78.6％（11/14）with VHA and 50.0％（5/10）with VA（*P*＝0.143）; MRD negativity rates were 64.3％（9/14）and 20.0％（2/10）, respectively（*P*＝0.032）. The main adverse events were myelosuppression, gastrointestinal reactions, and infections during neutropenia. Rates of grade 3–4 neutropenia and decreased hemoglobin were similar between groups, whereas grade 3–4 thrombocytopenia was more frequent with VHA than with VA（76.9％ *vs* 45.5％, *P*＝0.015）. With a median follow-up of 13 months（range, 1–59）, 1 year RFS was 69.9％（95％ *CI*: 53.1％–92.2％）with VHA and 55.6％（95％ *CI*: 40.1％–77.1％）with VA（*P*＝0.305）. The 1 year OS rates were 91.7％（95％ *CI*: 77.3％–100.0％）and 58.2％（95％ *CI*: 41.7％–81.4％）, respectively（*P*＝0.024）. Among high risk patients, 1 year RFS and OS were higher with VHA than with VA（RFS: 66.2％ *vs* 37.5％, *P*＝0.046; OS: 85.7％ *vs* 48.0％, *P*＝0.011）. Undergoing allogeneic hematopoietic stem cell transplantation（allo-HSCT）significantly improved RFS and OS（*P*＝0.027 and 0.047, respectively）. On multivariable analysis, ELN risk classification and MRD negativity after the first cycle were independent prognostic factors for RFS. Treatment regimen（VHA *vs* VA）, MRD negativity after the first cycle, and receipt of transplantation were independent prognostic factors for OS.

**Conclusion:**

VHA provides clinical benefit in newly diagnosed AML patients who are unfit for intensive chemotherapy and in older adults, with particularly favorable outcomes in high risk patients; sequential allo-HSCT confers additional benefit, and associated adverse events are manageable.

急性髓系白血病（AML）具有较强的异质性，随着分子靶向治疗新药及方案的涌现，治疗效果显著改善。然而，部分初诊因合并症不适合强诱导治疗或老年AML患者，由于高强度治疗的耐受性差、诱导治疗剂量不足等因素，疗效不佳、生存期短、复发率高。多项研究表明Bcl-2抑制剂联合去甲基化药物阿扎胞苷（AZA）对于老年及初治不适合高强度治疗的AML疗效确切[1]，但在临床中仍有约30％的患者疗效不佳。高三尖杉酯碱（HHT）在难治性AML再诱导治疗中疗效肯定，不良反应较少。国内对于HHT联合维奈克拉（VEN）、AZA（VHA）方案治疗初诊AML疗效及安全性的研究报道较少。我们对本中心接受VHA和VA方案诱导治疗的初诊不适合强化疗及老年AML患者进行回顾性分析和比较，以观察其有效性、安全性以及生存的影响因素，探讨VA方案中添加HHT是否提高初治患者疗效。

## 病例与方法

1. 病例资料：本研究回顾性分析2018年9月至2021年7月郑州大学附属肿瘤医院收治的59例初诊AML患者的临床资料，所有患者根据细胞形态学、免疫学、细胞遗传学、分子生物学（MICM）分型确诊。以美国东部肿瘤协作组（ECOG）标准进行体能评分。以欧洲白血病网（ELN）标准进行预后危险分层。所有患者根据情况均采用VHA或VA方案诱导治疗。

2. 治疗方案：VHA方案组：VEN 100 mg第1天，200 mg第2天，300 mg第3天，400 mg第4～28天，口服，每日1次（VEN用量根据用药期间耐受性、合并用药及骨髓抑制情况调整）；AZA 75 mg·m^−2^·d^−1^，第1～7天；HHT 1 mg/d，静脉滴注，第1～7天。VA方案组：VEN 100 mg第1天，200 mg第2天，300 mg第3天，400 mg第4～28天，口服，每日1次（VEN用量根据用药期间耐受性、合并用药及骨髓抑制情况调整）；AZA 75 mg·m^−2^·d^−1^，第1～7天。采用伏立康唑及泊沙康唑预防及治疗真菌感染时，调整VEN至100 mg，口服，每日1次。若出现Ⅳ度骨髓抑制，同时合并重症感染时，可停用VEN。治疗第14天复查骨髓，若原始细胞<5％，结合骨髓抑制情况，调整继续口服或停用VEN；若下降比例>50％但未达<5％，继续口服VEN；同时有检测条件的单位应检测VEN血药浓度并依据调整剂量。巩固方案包括VA、VHA、中剂量Ara-C（1.5 g·m^−2^·d^−1^，每12 h 1次，静脉滴注，第1～3天）、HA（HHT 2.5 mg·m^−2^·d^−1^，静脉滴注，第1～4天；阿糖胞苷100 mg·m^−2^·d^−1^，静脉滴注，第1～7天）、DA（柔红霉素60 mg·m^−2^·d^−1^，静脉滴注，第1～3天；阿糖胞苷100 mg·m^−2^·d^−1^，静脉滴注，第1～7天）等。无效患者采用D-CAG、D-CHAG、IA等方案再诱导治疗。达完全缓解（CR）后按照2017 ELN危险度分层选择不同的后续治疗方案，低危组与无合适供者的中、高危组患者进行巩固化疗，有合适供者的中、高危组患者选择接受异基因造血干细胞移植（allo-HSCT）治疗。

3. 治疗期间不良事件的发生及处理原则：按照NCI常规不良事件标准4.0分级标准评定不良反应。治疗期间定期监测血常规和肝肾功能，针对药物引起的骨髓抑制，采取输注相应血制品及使用促造血生成药物。当患者出现发热或寒战合并感染时，常规进行血培养及病毒检测，并按经验予以广谱抗生素治疗，同时予以泊沙康唑（5 ml/次，每日4次，间隔6 h）预防真菌感染，常规抗生素治疗5～7 d无效或者CT提示真菌感染者给予静脉抗真菌治疗。

4. 观察指标及疗效评价：治疗前行骨髓穿刺完善MICM分型，诱导化疗结束后复查骨髓涂片及微小残留病（MRD）评估疗效。疗效指标分为完全缓解（CR）、CR伴血细胞不完全恢复（CRi）、部分缓解（PR）和未缓解（NR），观察患者复合缓解率（CR率+CRi率）、总有效率（ORR）、总生存（OS）期及无复发生存（RFS）期。

5. 随访：存活病例随访至2024年4月20日，死亡病例随访至死亡日期，中位随访时间为13（1～59）个月。随访方式包括电话随访、门诊复查、查阅病历，所有患者均未失访。

6. 统计学处理：应用SPSS 23.0软件进行统计学分析。计数资料组间比较采用Fisher确切概率法，计量资料组间比较采用独立样本*t*检验；采用Kaplan-Meier法和Cox回归模型进行生存预后的单因素及多因素分析。*P*<0.05为差异有统计学意义。

## 结果

1. 患者基本临床资料：共纳入59例初治AML患者，其中男25例，女34例，中位年龄63（13～86）岁。参考ELN分层，高危组24例（40.7％），中危组31例（52.5％），低危组4例（6.8％）。予以VHA方案诱导治疗26例，VA方案诱导治疗33例。VHA组、VA组在ELN分层中、高危患者比例差异有统计学意义，两组患者的年龄、ECOG评分及基因突变等其他特征差异均无统计意义（均*P*>0.05）。所有患者中突变比例较高的基因分别为RUNX1（11例，18.6％）、NPM1（10例，16.9％）、FLT3-ITD（10例，16.9％）、IDH1/IDH2（13例，22％）、TP53（5例，8.5％），ASXL1（5例，8.5％）（表1）。

**表1:** VHA组和VA组患者临床特征比较

临床特征	VHA组（26例）	VA组（33例）	统计量	*P*值
年龄［岁，*M*（范围）］	55（13～73）	68（54～91）	3.639	0.061
性别［例（％）］			1.146	0.284
男	9（34.6）	16（48.5）		
女	17（65.3）	17（51.5）		
ECOG评分［例（％）］			5.213	0.157
0～1分	24（92.3）	25（75.8）		
≥2分	2（7.7）	8（24.2）		
ELN预后分层［例（％）］			6.378	0.041
低危	3（11.5）	1（3.0）		
中危	9（34.6）	22（66.7）	5.376	0.020
高危	14（53.8）	10（30.3）	3.340	0.068
基因突变［例（％）］				
FLT3-ITD	3（11.5）	7（21.2）	0.967	0.325
NPM1	5（19.2）	5（15.2）	0.172	0.678
TP53	1（3.8）	4（12.1）	1.284	0.257
IDH1/IDH2	5（19.2）	8（24.2）	0.213	0.645
ASXL1	4（15.4）	1（3.0）	2.862	0.091
RUNX1	5（19.2）	6（18.2）	0.011	0.918
序贯巩固治疗［例（％）］	23（88.4）	30（90.9）	0.095	0.757
巩固治疗疗程［个，*M*（范围）］	5（2～8）	6（1～10）	0.028	0.968
序贯移植［例（％）］	5（19.2）	0（0）	6.934	0.008

**注** VHA：维奈克拉+阿扎胞苷+高三尖杉酯碱；VA：维奈克拉+阿扎胞苷

2. 诱导治疗缓解率及疗效：VHA方案组VEN诱导治疗中位用药时间15（10～28）d，ORR率88.4％（23/26），1个疗程复合缓解率为80.8％（21/26），1个疗程MRD转阴率73.1％（19/26）；VA方案组VEN诱导治疗中位用药时间21（14～28）d，ORR率90.9％（30/33），1个疗程复合缓解率为75.8％（25/33），1个疗程MRD转阴率60.6％（20/33），两组间比较差异均无统计学意义（*OR*＝1.818，95％ *CI*：0.369～8.959，*P*＝0.458；*OR*＝0.744，95％ *CI*：0.211～2.620，*P*＝0.645；*OR*＝0.567，95％ *CI*：0.186～1.725，*P*＝0.315）。

对于高危组患者，VHA方案组和VA方案组的1个疗程ORR分别为78.6％（11/14）和50.0％（5/10），差异无统计学意义（*OR*＝0.273，95％ *CI*：0.046～1.616，*P*＝0.143）；1个疗程MRD转阴率分别为64.3％（9/14）和20.0％（2/10），差异有统计学意义（*OR*＝0.139，95％ *CI*：0.021～0.925，*P*＝0.032）。接受allo-HSCT的5例患者，其中高危组4例，中危组1例，移植时，3例处于CR，2例处于NR，3例为亲缘单倍体移植，1例为同胞全相合移植，1例为非血缘allo-HSCT。

对于不同年龄的患者进行分析，VHA方案组<60岁患者1个疗程的CR率、MRD转阴率高于> 60岁患者［93.8％（15/16）对60.0％（6/10），*OR*＝0.105，95％ *CI*：0.009～1.088，*P*＝0.034；87.5％（14/16）对50.0％（5/10），*OR*＝0.143，95％ *CI*：0.021～0.986，*P*＝0.036）。VA方案组差异无统计学意义。

对于携带FLT3突变、NPM1突变、IDH突变、RUNX1突变患者，VHA方案组和VA方案组1个疗程缓解及MRD转阴情况见表2。

**表2:** VHA和VA方案在伴不同突变急性髓系白血病患者中的疗效（例）

组别	例数	1个疗程缓解	1个疗程MRD转阴
伴FLT3突变			
VHA方案组	3	3	2
VA方案组	7	6	4
伴NPM1突变			
VHA方案组	5	4	3
VA方案组	5	5	5
伴IDH突变			
VHA方案组	5	5	3
VA方案组	8	8	8
伴RUNX1突变			
VHA方案组	5	5	3
VA方案组	6	5	3

**注** VHA：维奈克拉+阿扎胞苷+高三尖杉酯碱；VA：维奈克拉+阿扎胞苷；MRD：微小残留病

通过纳入年龄、FLT3-ITD突变、NPM1突变、ELN分层对两个方案组分别进行单因素分析，结果显示：VHA方案组年龄小于60岁与患者1个疗程CR率及MRD转阴率相关；VA方案组ELN分层与1个疗程MRD转阴率相关。

3. 长期疗效及生存情况：截至2024年4月20日（死亡病例截至死亡日期），所有患者中位随访时间为13（1～59）个月，VHA方案组中位随访时间为11（1～29）个月，VA方案组中位随访时间为14（1～59）个月。随访期内，VHA和VA方案组1年RFS率分别为69.9％（95％ *CI*：53.1％～92.2％）和55.6％（95％ *CI*：40.1％～77.1％），两组差异无统计学意义（*P*＝0.305）；1年OS率分别为91.7％（95％ *CI*：77.3％～100.0％）和58.2％（95％ *CI*：41.7％～81.4％），两组差异有统计学意义（*P*＝0.024）（图1）。

**图1:**
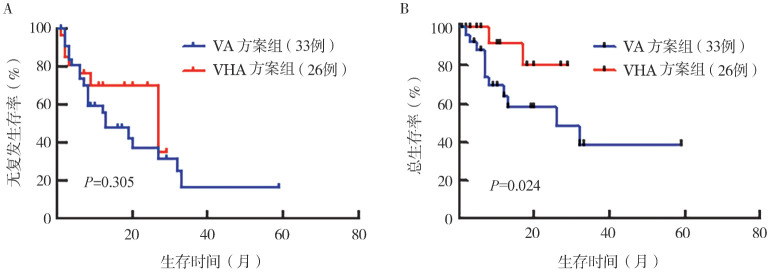
采用VHA及VA方案诱导治疗的初诊急性髓系白血病患者的无复发生存（A）和总生存（B）比较 **注** VHA：维奈克拉+高三尖杉酯碱+阿扎胞苷；VA：维奈克拉+阿扎胞苷

对于高危组患者分析显示，VHA方案组的1年RFS率及OS率显著高于VA方案组（RFS：66.2％对37.5％，*P*＝0.046；OS：85.7％对48.0％，*P*＝0.011）。5例VHA方案组患者行allo-HSCT，尤其对于高危组患者，接受allo-HSCT患者能够明显提高OS率及RFS率（*P*值分别为0.027、0.047）。

通过纳入年龄、ELN分层、不同用药方案、1个疗程CR、1个疗程MRD转阴、是否移植等多因素分析发现，ELN分层（*HR*＝2.171，95％ *CI*：1.029～4.582，*P*＝0.042）、1个疗程MRD转阴（*HR*＝5.254，95％ *CI*：2.03～13.601，*P*＝0.001）与RFS延长相关。不同用药方案（*HR*＝2.322，95％ *CI*：1.082～4.985，*P*＝0.031）、1个疗程MRD转阴（*HR*＝3.402，95％ *CI*：1.504～7.693，*P*＝0.003）、是否移植（*HR*＝0.276；95％ *CI*：0.08～0.911，*P*＝0.035）与OS延长相关。

4. 不良反应：两种方案最常见不良反应均为Ⅲ～Ⅳ度血液学不良反应，VHA方案组严重粒细胞缺乏（80.8％对57.6％，*P*＝0.058）、血红蛋白减少（73％对60.6％，*P*＝0.172）发生率较VA方案组差异无统计学意义。VHA方案组严重血小板减少发生率高于VA方案组（76.9％对45.5％，*P*＝0.015）。VHA方案组和VA方案组粒细胞缺乏恢复的中位时间差异无统计学意义（11.96 d对8.70 d，*P*＝0.844）；血小板减少恢复的中位时间差异无统计学意义（18.46 d对13.90 d，*P*＝0.606）。两组患者最常见的非血液学不良反应包括胃肠道反应、粒细胞缺乏期发热，差异均无统计学意义（*P*>0.05）。两组患者治疗过程中均未出现重症感染及肿瘤溶解综合征。

## 讨论

尽管近年来在AML分子靶向治疗方面取得诸多进展，但仍存在初治疗效欠佳及耐药导致疾病进展的患者，其应用传统化疗治愈率不足10％，CR率36％[2]–[3]，中位生存期短；除此之外，初治不适合强化疗及老年AML患者，由于存在对常规诱导化疗耐受性差及持续缓解时间短等因素，长期疗效欠佳，采用去甲基化药物及低剂量化疗的有效率仅为40％，中位OS期不足1年[2]。对于多数疗效欠佳及复发难治AML患者，allo-HSCT是获得长期生存的唯一手段，但移植前骨髓缓解深度直接影响移植后长期生存状态[4]。因此，选择何种方案提高初诊不耐受强化疗及老年AML患者的疗效成为当务之急。

Bcl-2作为一种抗凋亡蛋白，通过选择性抑制抗凋亡蛋白Bcl-2，激活线粒体通路，诱导细胞凋亡。目前多项临床研究表明Bcl-2抑制剂联合AZA对于老年及初治不适合强化疗AML患者安全有效[5]，对于复发难治患者，有小样本研究提示CR率也能达32％～50％[1],[6]，但仍存在30％患者由于特殊基因特征等因素对于Bcl-2抑制剂联合AZA疗效欠佳，无法受益于此方案。目前，对于Bcl-2抑制剂联合AZA疗效欠佳的具体机制尚不清楚，既往研究显示可能与Bcl-2家族蛋白Mcl-1以及Bcl-XL上调等多种因素相关[7]。Bcl-2抑制剂虽减少了Bim与Bcl-2的结合，但却代偿性增加Bim与Mcl-1结合，从而导致凋亡蛋白Mcl-1上调，从而抑制Bcl-2抑制剂诱导细胞凋亡。因此，选择性抑制Mcl-1表达及活性具有潜在治疗价值[8]。

HHT作为一种从三尖杉植物中萃取的生物碱，国内外多项研究表明其通过联合其他化疗药物起到抗白血病效应[9]–[10]，虽然作用机制尚不清楚，有研究报道可能通过抑制蛋白质合成及促进细胞凋亡实现对白血病细胞的抑制和杀伤，在细胞凋亡通路蛋白变化之前就降低Mcl-1及c-myc的表达，并通过抑制蛋白合成及表达，从而影响白血病细胞生存；HHT也可通过上调Bax、Bid等促凋亡蛋白表达，从而引起细胞凋亡[11]–[12]。此外，临床前研究证明HHT可增强VEN（无论是否联合AZA）的抗白血病作用[13]–[14]。临床应用中，尤其在难治复发AML患者中，VHA方案的反应率高于VA方案[14]。

基于以上研究背景及Bcl-2抑制剂、AZA及HHT药物作用特点，对于初诊AML，我们尝试予以HHT联合Bcl-2抑制剂及AZA协同治疗，观察其在初诊不适合强化疗及老年AML患者中的有效性、安全性及对长期生存的影响。

本研究纳入59例初诊AML患者，研究结果显示，VHA方案在ORR率、复合缓解率及MRD转阴率方面与VA方案相当。尤其对于高危组患者，虽然在ORR率、复合缓解率差异无统计学意义，但从趋势及MRD转阴率［64.3％（9/14）和20％（2/10），*P*＝0.032］看，HHT可增强VA方案的抗白血病作用。年龄因素方面，对于<60岁患者，VHA方案较VA方案表现出更高的CR率及MRD转阴率，VHA研究队列偏年轻，可能影响其对疗效影响的分析。对于携带FLT3-ITD突变患者，两个方案组均获得不错疗效，可能与加用索拉非尼及样本量有限有关。对于携带NPM1及IDH、RUNX1、TP53、ASXL1突变患者，两个方案组疗效相当，并且不同突变亚型间疗效相当，由于样本量有限，未进行统计学分析。

此外，两组缓解患者均采用巩固治疗，虽然随访时间有限，1年RFS率VHA组与VA组差异无统计学意义，但在1年OS率及高危组患者1年OS率、RFS率方面，VHA方案体现出一定优势，结合Cox多因素综合分析，这也可能与VHA方案高危组患者更多接受allo-HSCT（OS率及RFS率*P*值分别为0.027、0.047）及VHA方案在MRD转阴率较VA方案略显优势（73.1％对60.6％）有关。另外，本研究中VHA方案在血液学及非血液学不良反应方面与VA方案相当，主要不良反应为骨髓抑制、恶心呕吐、发热、乏力等，未出现重症感染，经过抗感染、输血、予以相应刺激因子及对症治疗后恢复。VHA方案Ⅲ～Ⅳ度粒细胞缺乏及血红蛋白减少发生率与VA方案相近，但Ⅲ～Ⅳ度严重血小板减少发生率高于VA方案，同时两组粒细胞缺乏及血小板减少恢复的中位时间相近。

综上所述，VHA方案总体耐受性良好，疗效与VA方案相当，尤其对于<60岁高危患者，提高了缓解深度及长期RFS率。但由于是回顾性研究，样本量较小，且随访时间短，结果会存在一定片面性，仍需后期扩大随访患者及延长随访时间，从而获得更多客观结论。

## References

[1] Lou Y, Shao L, Mao L (2020). Efficacy and predictive factors of venetoclax combined with azacitidine as salvage therapy in advanced acute myeloid leukemia patients: A multicenter retrospective study[J]. Leuk Res.

[2] Fiorentini A, Capelli D, Saraceni F (2020). The Time Has Come for Targeted Therapies for AML: Lights and Shadows[J]. Oncol Ther.

[3] National Cancer Institute (2021). Surveillance, epidemiology, and end results program[M].

[4] Thol F, Heuser M (2021). Treatment for relapsed/refractory acute myeloid leukemia[J]. Hemasphere.

[5] DiNardo CD, Pratz K, Pullarkat V (2019). Venetoclax combined with decitabine or azacitidine in treatment-naive, elderly patients with acute myeloid leukemia[J]. Blood.

[6] Aldoss I, Yang D, Pillai R (2019). Association of leukemia genetics with response to venetoclax and hypomethylating agents in relapsed/refractory acute myeloid leukemia[J]. Am J Hematol.

[7] Chiron D, Dousset C, Brosseau C (2015). Biological rational for sequential targeting of Bruton tyrosine kinase and Bcl-2 to overcome CD40-induced ABT-199 resistance in mantle cell lymphoma[J]. Oncotarget.

[8] Jin S, Cojocari D, Purkal JJ (2020). 5-Azacitidine Induces NOXA to Prime AML Cells for Venetoclax-Mediated Apoptosis[J]. Clin Cancer Res.

[9] Feldman E, Arlin Z, Ahmed T (1992). Homoharringtonine in combination with cytarabine for patients with acute myelogenous leukemia[J]. Leukemia.

[10] Wu LY, Li X, Su JY (2011). Efficacy and safety of CHG regimen (low-dose cytarabine,homoharringtonine with G-CSF priming) as induction chemotherapy for elderly patients with high-risk MDS or AML transformed from MDS[J]. J Cancer Res Clin Oncol.

[11] Tang R, Faussat AM, Majdak P (2006). Semisynthetic homoharringtonine induces apoptosis via inhibition of protein synthesis and triggers rapid myeloid cell leukemia-1 down-regulation in myeloid leukemia cells[J]. Mol Cancer Ther.

[12] Yin S, Wang R, Zhou F (2011). Bcl-xL is a dominant antiapoptotic protein that inhibits homoharringtonine-induced apoptosis in leukemia cells[J]. Mol Pharmacol.

[13] Sivakolundu SG, Mabrouk PA (2003). Structure-function relationship of reduced cytochrome c probed by complete solution structure determination in 30％ acetonitrile/water solution[J]. J Biol Inorg Chem.

[14] Jin H, Zhang Y, Yu S (2023). Venetoclax Combined with Azacitidine and Homoharringtonine in Relapsed/Refractory AML: A Multicenter, Phase 2 Trial[J]. J Hematol Oncol.

